# Structural Conservation and Assembly Plasticity of a Divergent Phenuivirus Nucleoprotein

**DOI:** 10.3390/life16101605

**Published:** 2026-09-25

**Authors:** Shan Du, Lixuan Qi, Wenni Zhang, Qianqian Shao, Qilu Weng, Runping Qin, Chang Liu, Zan Li, Litao Sun

**Affiliations:** 1 School of Public Health (Shenzhen), Shenzhen Campus of Sun Yat-sen University, Sun Yat-sen University, Shenzhen 518107, China; dush6@alumni.sysu.edu.cn (S.D.); qilx3@mail2.sysu.edu.cn (L.Q.); zhangwn28@mail2.sysu.edu.cn (W.Z.); shaoqq@mail2.sysu.edu.cn (Q.S.); wengqlu@alumni.sysu.edu.cn (Q.W.); qinrp3@mail2.sysu.edu.cn (R.Q.); liuch686@mail2.sysu.edu.cn (C.L.); 2 Shenzhen Key Laboratory of Pathogenic Microbes and Biosafety, School of Public Health (Shenzhen), Sun Yat-sen University, Shenzhen 518107, China

**Keywords:** *Phenuiviridae*, nucleoprotein, RNA association, oligomerization, suramin

## Abstract

Tick-borne phenuiviruses include a diverse set of emerging pathogens whose nucleoproteins (NPs) package viral RNA despite extensive sequence divergence across lineages. We examined the NP of Tacheng tick virus 2 (TcTV2), a highly divergent member of the genus *Uukuvirus*. Phylogenetic analysis of an expanded NP dataset supported the placement of TcTV2 within the broader *Uukuvirus* lineage, and the AlphaFold model predicted a bilobed core with an electropositive groove resembling those of other phenuivirus NPs. Structural comparisons of the predicted model indicated the greatest variation in the peripheral regions. Recombinant TcTV2 NP co-purified with bacterial RNA and formed heterogeneous ring-like particles, with pentamer-like particles predominating in cryo-EM 2D classes. Truncation of the distal N-terminal region (NP_152–440_) did not abolish ring-like particle formation under the tested conditions. Suramin altered the thermal denaturation profile of TcTV2 NP and produced concentration-dependent responses on an NP-loaded BLI sensor in exploratory measurements. Molecular docking and a single 100-ns molecular dynamics simulation suggested a possible suramin-binding mode near the electropositive groove. TcTV2 NP therefore combines a predicted phenuivirus-like core with experimentally observed RNA association and heterogeneous ring-like particle formation, while the computational analyses nominate the electropositive groove as a candidate surface for further ligand interaction studies.

## 1. Introduction

The family *Phenuiviridae* encompasses genetically diverse viruses with growing relevance to human and animal health. Its members include tick-borne viruses such as severe fever with thrombocytopenia syndrome virus (SFTSV) and Tacheng tick virus 2 (TcTV2), an emerging member of the genus *Uukuvirus*, as well as the mosquito-borne Rift Valley fever virus (RVFV) [1,2]. First identified in northwestern China, TcTV2 was subsequently detected in ticks, wildlife, livestock, and humans in neighboring regions and, more recently, in Türkiye [3,4,5]. Uukuniemi virus (UUKV), the prototype of the genus *Uukuvirus*, has long served as an experimental model for studying tick-borne phenuivirus entry, assembly, and replication [6,7]. Several tick-borne phenuiviruses have been associated with human infection, but the molecular events governing genome packaging and replication have not been defined for many recently identified lineages.

The nucleoprotein (NP) performs several functions during bunyaviral replication. It binds and protects the viral RNA genome, organizes ribonucleoprotein (RNP) complexes, and presents the encapsidated RNA to the viral polymerase during transcription and replication [8,9,10,11,12,13,14,15]. To perform these tasks, NP must bind RNA while forming reversible contacts with neighboring NP molecules. These contacts shape the RNP and regulate access to the viral genome. Phenuivirus nucleoproteins (NPs) show considerable sequence divergence and differ in their terminal regions and oligomeric arrangements, raising the question of which structural features preserve RNA encapsidation across lineages.

Structures of representative bunyaviral NPs share a charged RNA-binding groove but differ in terminal-arm organization and oligomeric state [16,17,18,19,20,21]. Flexible terminal elements allow these proteins to form alternative ring-like and helical arrangements [22,23,24,25,26]. Mutational studies have also identified positively charged RNA-contacting residues and NP–NP interaction surfaces that contribute to NP function and viral replication [27,28,29,30]. These studies define conserved RNA-binding and oligomerization features in several representative phenuivirus NPs, but whether the same structural organization is retained in highly divergent uukuvirus NPs has not been established.

Comparisons among orthobunyavirus, nairovirus, and hantavirus NPs have uncovered distinct folds, oligomeric interfaces, and RNP arrangements across bunyaviral lineages [31,32,33,34,35]. TcTV2 NP has not been experimentally characterized with respect to its structural organization, nucleic-acid association, or higher-order assembly. Its sequence divergence offers a direct test of whether a phenuivirus-like RNA-binding core can persist alongside substantial changes in peripheral regions and oligomeric organization.

Structure prediction, when evaluated alongside experimental data, can help reveal evolutionary relationships among the NPs of negative-sense RNA viruses [36]. We combined structural modeling, biochemical analysis, electron microscopy, truncation experiments, and ligand-binding measurements to examine the predicted structural organization, RNA association, and ring-like particle formation of TcTV2 NP. The electropositive groove predicted in the TcTV2 NP model provided a candidate surface for examining the association of a polyanionic ligand. Suramin was included as a model polyanionic ligand because it has been reported to bind the RNA-binding cavity of SFTSV NP [19].

## 2. Materials and Methods

### 2.1. Sequence Analysis, Structural Prediction, and Visualization

The dataset comprised 30 phenuivirus NP amino acid sequences, including close relatives of TcTV2 NP, broader representatives of the genus *Uukuvirus*, and selected representatives of other *phenuivirus* lineages. Changping tick virus 1, Dermacentor reticulatus uukuvirus, Qinghai Lake Virus 3, Meitian tick virus, and American dog tick virus were included (sequence names and accession numbers are provided in Table S1). Markedly incomplete or redundant sequences and records lacking sufficient NP annotation were excluded. Sequences were aligned using MAFFT v7.525 with the L-INS-i strategy [37]. The complete alignment contained 563 columns. For phylogenetic analysis, columns with less than 50% non-gap occupancy were removed, yielding an alignment of 293 positions; remaining gaps were treated as missing or unknown characters. Maximum-likelihood analysis was performed using IQ-TREE v3.1.3 under the Q.PFAM + I + G4 model, selected using ModelFinder as implemented in IQ-TREE v3.1.3 based on the Bayesian Information Criterion (BIC) [38,39]. Branch support was assessed using 1000 SH-aLRT replicates and 1000 ultrafast bootstrap replicates [38,40]. No biological outgroup was imposed; midpoint rooting was used only for graphical display. Pairwise amino-acid similarity was calculated from the complete, untrimmed alignment. For each sequence pair, columns containing a gap in either sequence were excluded. Similarity was defined as the percentage of identical or BLOSUM62-positive residue pairs among the remaining pairwise comparable positions [41].

The predicted monomeric TcTV2 NP structure was retrieved from the AlphaFold Protein Structure Database (https://alphafold.ebi.ac.uk/; entry AF-0000000365807691 [42], accessed on 21 September 2026), with local prediction confidence assessed by pLDDT scores. A hypothetical pentameric arrangement was generated independently using AlphaFold 3 through the AlphaFold Server [43], with five copies of the full-length TcTV2 NP sequence as input. Model 0 from the TcTV2 NP 5-mer job was selected for visualization and interface analysis. Model confidence was evaluated using mean Cα pLDDT, pTM, ipTM, pairwise ipTM, and predicted aligned error values, as summarized in Table S4. Structural models were visualized in PyMOL v3.0.3 (Schrödinger, LLC, New York, NY, USA) [44] and UCSF ChimeraX v1.10 (University of California, San Francisco, CA, USA) [45]. Structural RMSDs were calculated in PyMOL using Cα atoms over the residue ranges specified in Table S2.

### 2.2. Expression and Purification of TcTV2 NP

The coding sequence of full-length TcTV2 NP (NCBI Reference Sequence: NC_055426.1) was codon-optimized for *Escherichia coli* and synthesized by General Biol Co., Ltd. (Chuzhou, China). The construct was cloned into pET-21a(+) (Novagen, Madison, WI, USA), and the C-terminal His tag was extended from the vector-derived 6 × His sequence to a 10 × His tag during construct preparation. The resulting plasmid was transformed into *Escherichia coli* BL21(DE3) cells. Cells were cultured in 1 L of LB medium containing 50 μg/mL ampicillin at 37 °C with shaking at 220 rpm. When the optical density at 600 nm (OD600) reached 0.6–0.8, protein expression was induced with 0.5 mM isopropyl-β-D-thiogalactopyranoside (IPTG) at 16 °C for 18 h.

Cells were harvested by centrifugation at 4200 rpm for 35 min at 4 °C. Cell pellets were resuspended in lysis buffer containing 20 mM Tris-HCl (pH 7.5), 300 mM NaCl, 5% glycerol, and 5 mM imidazole, and disrupted using a high-pressure homogenizer. PMSF was added to a final concentration of 1 mM immediately before cell disruption. Cells were disrupted twice at 1500 bar using a JN-Mini Pro low-temperature high-pressure homogenizer (Guangzhou Juneng Nano & Biotechnology Co., Ltd., Guangzhou, China). The lysate was clarified by centrifugation at 20,000 rpm for 30 min at 4 °C using an Optima XPN-100 ultracentrifuge equipped with a Type 70 Ti rotor (Beckman Coulter Life Sciences, Indianapolis, IN, USA).

After centrifugation, the soluble fraction was loaded onto Ni-NTA resin. The resin was washed stepwise with buffers containing increasing imidazole concentrations from 20 to 100 mM to remove nonspecifically bound proteins. TcTV2 NP was then eluted with buffer containing 500 mM imidazole. The eluate was further purified by size-exclusion chromatography (SEC) on a Superose 6 Increase 10/300 GL column (Cytiva, Marlborough, MA, USA) equilibrated with 20 mM Tris-HCl (pH 7.5), 300 mM NaCl, and 2 mM DTT. Peak fractions were examined by SDS-PAGE. Full-length TcTV2 NP was concentrated using a 30 kDa MWCO Amicon Ultra centrifugal filter (MilliporeSigma, Burlington, MA, USA), whereas NP152–440 was concentrated using a 10 kDa MWCO filter. Purified proteins were stored at −80 °C.

The N-terminally truncated construct NP_152–440_ was expressed and purified using the same affinity chromatography and SEC procedures. Additional truncation constructs were examined only during expression and solubility screening because they did not yield sufficient soluble protein for further characterization.

### 2.3. Characterization of Co-Purified Nucleic Acid

Purified TcTV2 NP at a final concentration of 1.0 mg/mL was incubated with RNase A or DNase I at final concentrations of 0.01, 0.025, 0.05, 0.1, or 1.0 mg/mL. For each enzyme concentration, digestion reactions were prepared independently in duplicate and incubated at 37 °C for 30 min. Untreated NP samples were processed in parallel as controls. The reaction mixtures were resolved on 1.0% (*w*/*v*) native agarose gels prepared in 1× TAE buffer. Electrophoresis was performed at a constant current of 30 mA for 30 min on ice. Nucleic acid was stained with GelRed (Biosharp, Beijing, China) and visualized using a UV transilluminator (ChemiDoc XRS+, Bio-Rad Laboratories, Inc., Hercules, CA, USA).

#### RNA Extraction, Library Preparation, and Sequence Analysis

RNA was extracted using TRIzol Reagent (Thermo Fisher Scientific, Waltham, MA, USA) from two sample types. The TcTV2 NP-RNA sample comprised RNA recovered from recombinant TcTV2 NP after SEC, whereas the TcTV2 NP-*E. coli* sample comprised total RNA extracted from TcTV2 NP-expressing *E. coli* cells and served as the input control. Both RNA preparations were treated with DNase I, recovered by ethanol precipitation, and submitted to RiboBio Co., Ltd. (Guangzhou, China) for library preparation and sequencing. Ribosomal RNA was depleted, and libraries were prepared using the NEBNext Ultra RNA Library Prep Kit for Illumina (New England Biolabs, Ipswich, MA, USA). Sequencing was performed on an Illumina NovaSeq 6000 platform using 150-bp paired-end reads.

Raw reads were processed using Trimmomatic v0.36 with a four-base sliding window requiring a mean quality score > 15; reads shorter than 35% of their original length were discarded [46]. Reads matching the RNAcentral rRNA reference database were removed using Bowtie2 v2.2.5 in sensitive mode [47,48]. The remaining reads were aligned to the *E. coli* BL21(DE3) reference genome assembly ASM1316701v1 (RefSeq accession GCF_013167015.1) using TopHat v2.0.13, allowing no more than two mismatches [49]. Uniquely mapped reads were categorized using the corresponding NCBI genome annotation. Upstream regions were defined from the annotated transcription start site to 1 kb upstream, whereas downstream regions were defined as the 1-kb regions upstream and downstream of the annotated transcription termination site.

One library was analyzed for each sample type. The sequencing results were therefore interpreted descriptively, without differential expression testing or statistical analysis of transcript-specific enrichment.

### 2.4. On-Column Denaturation/Refolding Assay

An on-column denaturation and refolding procedure adapted from Toscana virus NP purification was used to remove co-purified bacterial RNA [24]. His-tagged TcTV2 NP was immobilized on Ni-NTA resin. The immobilized protein was treated with one column volume of denaturation buffer containing 20 mM Tris-HCl (pH 7.5) and 8 M urea for 10 min at 4 °C. The resin was washed extensively with high-salt buffer containing 20 mM Tris-HCl (pH 7.5) and 500 mM NaCl to remove residual nucleic acid and allow protein refolding. TcTV2 NP was subsequently eluted and further analyzed by SEC with continuous monitoring of absorbance at 260 and 280 nm.

### 2.5. Negative-Stain Electron Microscopy

Purified full-length TcTV2 NP and NP_152–440_ were diluted to 0.05 mg/mL in SEC buffer and applied to glow-discharged, carbon-coated copper grids. After incubation for 1 min, the grids were stained with 2% (*w*/*v*) uranyl acetate and air-dried. Images were acquired on a JEM-1400 Flash transmission electron microscope (JEOL Ltd., Tokyo, Japan) operated at 120 kV and equipped with a Morada G3 CCD camera. For the full-length TcTV2 NP dataset, 194 micrographs were collected at a nominal magnification of 40,000×, corresponding to a pixel size of 3.33 Å/pixel. Contrast transfer function parameters were estimated using CTFFIND4 v4.1.14 [50]. A total of 99,932 particles were automatically picked using the template-free Laplacian-of-Gaussian algorithm implemented in RELION 3.1 [51] and subjected to two-dimensional classification. NP_152–440_ was examined qualitatively by negative-stain EM and was not subjected to the same quantitative particle classification workflow.

### 2.6. Cryo-Electron Microscopy

A 3.5 μL aliquot of purified TcTV2 NP was applied to a glow-discharged Quantifoil R1.2/1.3 holey carbon grids coated with home-prepared graphene oxide. The grids were plunge-frozen in liquid ethane using a Vitrobot Mark IV (Thermo Fisher Scientific, Waltham, MA, USA) at 100% humidity, with a blot time of 3.5 s and a blot force of 0.

Cryo-EM data were collected on a Talos F200C (Thermo Fisher Scientific, Waltham, MA, USA) transmission electron microscope operated at 200 kV and recorded using a Falcon IV direct electron detector (Thermo Fisher Scientific, Waltham, MA, USA). A total of 422 movie stacks were acquired at a nominal magnification of 120,000×, corresponding to a calibrated pixel size of 1.185 Å/pixel, with a cumulative electron dose of approximately 60 e^−^/Å^2^.

Beam-induced motion correction was performed using MotionCor2 v1.5.0 [52]. Contrast transfer function (CTF) parameters were estimated using CTFFIND4 [50]. An initial set of particles was picked using the template-free LoG algorithm in RELION 3.1 [51]. However, particles picked by this approach failed to generate 2D class averages with recognizable structural features. Therefore, automatic particle picking approach was performed using EMAN2 v2.99 [53], with the previously reported RVFV RNP hexamer and pentamer structures (PDB: 4V9E and 4H5O, respectively) [17] used as references. To minimize potential model bias introduced by the references, both structures were low-pass filtered to 20 Å before particle picking. A total of 127,674 particles were initially picked and subsequently cleaned by reference-free 2D classification using RELION 3.1 [51]. All 2D classifications were performed without imposed rotational symmetry. Two ring-like classes comprising 1990 particles in total were selected and subjected to a second round of reference-free 2D classification. This yielded pentamer-like, hexamer-like, heptamer-like, and undefined classes.

### 2.7. Thermal Shift Assay

The effect of suramin on the thermal stability of TcTV2 NP was assessed by differential scanning fluorimetry using the Protein Thermal Shift™ Kit (Applied Biosystems, Thermo Fisher Scientific, Waltham, MA, USA). Each reaction was prepared in a final volume of 20 μL containing 14 μL assay buffer (20 mM HEPES, pH 7.5, and 150 mM NaCl), 2 μL Protein Thermal Shift dye (125×), 2 μL purified TcTV2 NP (2 mg/mL), and 2 μL suramin (MedChemExpress, Monmouth Junction, NJ, USA) at a final concentration of 10 μM. NP without suramin and suramin plus dye without protein were included as controls.

After incubation at room temperature for 30 min, fluorescence was monitored from 25 °C to 95 °C at a heating rate of 0.5 °C/min using a StepOnePlus™ Real-Time PCR System (Applied Biosystems, Thermo Fisher Scientific, Waltham, MA, USA). Melting temperatures (Tm) were calculated using Protein Thermal Shift Software v1.4 (Applied Biosystems, Thermo Fisher Scientific), and the thermal shift (ΔTm) was determined relative to the suramin-free control. The assay was performed in three independent experiments, with TcTV2 NP alone and TcTV2 NP plus suramin analyzed in parallel in each experiment.

### 2.8. Biolayer Interferometry

The interaction between suramin and TcTV2 NP was measured by biolayer interferometry using an Octet R8 system (Sartorius Lab Instruments GmbH & Co. KG, Göttingen, Germany). Purified His-tagged TcTV2 NP was immobilized on Ni-NTA biosensors. Measurements were performed in 200 μL volumes at 37 °C with shaking at 1000 rpm in assay buffer containing 20 mM Tris-HCl (pH 7.5), 100 mM NaCl, 2 mM DTT, and 0.05% (*v*/*v*) Tween-20. The loaded biosensors were transferred into twofold serial dilutions of suramin ranging from 1.25 to 20 μM. Each cycle comprised a 60-s baseline, a 300-s association step, and a 500-s dissociation step in assay buffer.

Sensorgrams were aligned to the mean response over the final 5 s of the baseline step, corrected at the start of dissociation, and processed using Savitzky–Golay filtering in Octet Analysis Studio v12.2.2.26. A buffer-only cycle recorded on each NP-loaded sensor was used for reference subtraction; no separate reference sensor was included. The association and dissociation phases were globally fitted using a 1:1 kinetic model, which was used empirically to summarize the concentration-dependent responses. Measurements were performed in two independent experiments. Additional control measurements were performed using an unloaded Ni-NTA sensor and an unrelated His-tagged protein-loaded Ni-NTA sensor, each exposed to 1.25–20 μM suramin. Neither control showed a conventional association–dissociation profile, and the control data were therefore not subjected to kinetic fitting. The fitted values are therefore reported as apparent KD values and are not interpreted as establishing binding stoichiometry or specificity.

### 2.9. Molecular Docking and Molecular Dynamics Simulation

The three-dimensional structure of suramin was obtained from PubChem (CID: 5361) and prepared using LigPrep (Schrödinger Release 2024-4; Schrödinger, LLC, New York, NY, USA) with the OPLS4 force field [54,55]. Protonation states were generated using Epik (Schrödinger Release 2024-4; Schrödinger, LLC, New York, NY, USA) over a pH range of 7.0 ± 2.0 [56,57]. The selected suramin state contained six deprotonated sulfonate groups and had a net formal charge of −6. The TcTV2 NP model from the AlphaFold Protein Structure Database (AF-0000000365807691) was prepared using the Schrödinger Protein Preparation Wizard [58]. Molecular docking was performed using Glide in standard precision (SP) mode [59]. The receptor grid was centered on the centroid of Thr250, Gly279, Val281, Thr287, Arg290, Phe358, Ser377, and Phe378 within the predicted electropositive groove, with inner and outer grid-box dimensions of 15 and 20 Å, respectively. Ten post-docking poses were minimized and ranked by GlideScore, and the top-ranked pose was selected for subsequent MD simulation.

A single 100-ns MD simulation of the TcTV2 NP–suramin complex was performed using GROMACS 2024.3 [60]. The protein was parameterized using the AMBER14SB force field, and suramin parameters were generated using GAFF2 and ACPYPE [61,62,63]. The complex was solvated with TIP3P water in an approximately 9.96-nm cubic box containing 0.15 M NaCl and counterions for charge neutralization. The system underwent 50,000 steps of steepest-descent energy minimization, followed by 50,000-step position-restrained NVT and NPT equilibration at 310 K and 1 bar. The production simulation was conducted under the NPT ensemble at 310 K and 1 bar using a 2.5-fs integration time step, with the Nosé–Hoover thermostat and Parrinello–Rahman barostat [64]. Long-range electrostatic interactions were treated using the particle mesh Ewald method, and the van der Waals cutoff was 1.2 nm. Protein–ligand interactions were analyzed using a 4.0-Å heavy-atom contact cutoff; hydrogen bonds were defined by a donor–acceptor distance of ≤3.5 Å and a donor–hydrogen–acceptor angle of ≥150°. Contact and hydrogen-bond occupancies were calculated over 1001 frames from the single trajectory. Molecular structures were visualized using UCSF ChimeraX v1.10 [45].

## 3. Results

### 3.1. The Predicted TcTV2 NP Model Contains a Phenuivirus-like RNA-Binding Core

Maximum-likelihood analysis of the expanded 30-sequence NP dataset placed TcTV2 within the broader *Uukuvirus* lineage of the family *Phenuiviridae* (Figure 1A). TcTV2 was recovered within a cluster containing Changping tick virus 1, Meitian tick virus, and Qinghai Lake Virus 3 with strong branch support (SH-aLRT/UFBoot, 100/100). The internal relationship specifying TcTV2 relative to the Meitian tick virus–Qinghai Lake Virus 3 pair received limited support (SH-aLRT/UFBoot, 10/53). Pairwise comparisons based on the complete, untrimmed multiple-sequence alignment showed that TcTV2 NP had the highest BLOSUM62-positive similarities to with Qinghai Lake Virus 3 (80.88%), Meitian tick virus (80.79%), and Changping tick virus 1 (78.31%) among the sequences analyzed (Figure 1B and Table S1).

The full-length monomeric TcTV2 NP model retrieved from the AlphaFold Protein Structure Database (model ID AF-0000000365807691) comprised an expanded N-arm (residues 1–211), an N-lobe (residues 212–310), and a C-lobe (residues 311–440) (Figure 1C). The N- and C-lobes formed a compact bilobed core. The N-arm was modeled in an extended arrangement and had lower prediction confidence than the core (Figure S2B). This confidence pattern is consistent with greater conformational variability but does not establish flexibility in solution. The expanded N-arm is used only as a topological description of the predicted model, not as evidence of a fixed conformation.

Superposition of the TcTV2 NP core with the corresponding regions of SFTSV, RVFV, and TOSV NPs yielded Cα RMSDs of 1.37, 1.07, and 1.02 Å, respectively. Full-length comparisons with more distantly related bunyaviral NPs identified substantial differences in overall fold and terminal organization (Figure 1D,E). The strongest structural correspondence mapped to helices α4–α5 and the bilobed RNA-binding core; the distal N-terminal region and preceding helical elements diverged more extensively (Figure 1F). The predicted TcTV2 NP model retains a phenuivirus-like core, while structural variation is concentrated in its peripheral elements.

### 3.2. Recombinant TcTV2 NP Co-Purifies with Bacterial RNA

SEC resolved TcTV2 NP into two partially overlapping species, designated P1 and P2 (Figure 2A(a)). Both peaks absorbed at 260 and 280 nm, and SDS-PAGE confirmed the presence of TcTV2 NP in each fraction. Peak-apex A260/A280 ratios were 1.26 for P1 and 1.29 for P2, consistent with co-purifying nucleic acid but insufficient to identify its chemical nature. Rechromatography of the predominant P2 fraction yielded another major P2 peak, demonstrating that this chromatographic state persisted under the experimental conditions (Figure 2A(b)).

On-column urea denaturation and refolding reduced the nucleic-acid-associated absorbance without eliminating either SEC species (Figure 2A(c)). P1 and P2 accounted for 14.9% and 85.1% of the relative peak areas, respectively, indicating only a modest change in their chromatographic distribution. The peak-apex A260/A280 ratio fell from 1.26 to 0.80 for P1 and from 1.29 to 0.67 for P2 (Figure 2B). Nucleic acid therefore contributed to the absorbance profiles of both species but did not alone account for their separation by SEC.

RNase A progressively depleted the NP-associated nucleic acid signal, whereas DNase I had comparatively little effect (Figure 2C). The co-purifying material was therefore predominantly RNA. Sequencing assigned most mapped reads to protein-coding regions of the *E. coli* genome (Table S3), identifying host–cell transcripts as the principal source of RNA recovered with recombinant TcTV2 NP. This association is consistent with retention of RNA-associated properties by the sequence-divergent NP, although it does not establish sequence-specific RNA binding.

### 3.3. TcTV2 NP Forms Heterogeneous Ring-like Particles

Because phenuivirus NPs characteristically assemble into higher-order complexes to encapsulate viral RNA, we sought to determine the oligomeric architecture of recombinant TcTV2 NP. Negative-stain electron micrographs of purified full-length TcTV2 NP contained abundant ring-like particles spanning a range of diameters (Figure 3A). Cryo-EM particle picking used templates derived from RVFV NP assemblies, after which the selected particles underwent reference-free two-dimensional classification without imposed rotational symmetry. The resulting classes comprised pentamer-like, hexamer-like, heptamer-like, and undefined particles (Figure 3B and Figure S2A). Of the 1990 retained particles, 1189 (59.7%) were pentamer-like, 492 (24.7%) were hexamer-like, 205 (10.3%) were heptamer-like, and 104 (5.3%) remained undefined. Because templates were used during particle picking, these frequencies describe the analyzed dataset rather than the oligomeric equilibrium in solution. Even within this constraint, several ring-like classes were recovered, with pentamer-like particles predominating. The limited number of particles and marked structural heterogeneity prevented high-resolution three-dimensional reconstruction.

An AlphaFold 3 model containing five full-length TcTV2 NP protomers adopted a closed-ring arrangement with a central pore (Figure 3C). Residues 1–100 were included in the prediction but omitted from the displayed model because of their low prediction confidence. The pentameric model had pTM and ipTM scores of 0.50 and 0.46, respectively, and was therefore treated as a model-derived hypothesis rather than an experimentally validated assembly. In the AF3-predicted pentamer model, residues within helices α3–α5 were positioned at the predicted protomer interface (Figure 3D). The model predicted interprotomer contacts between Ser139–Gln209, Gln164–Ala231, Met167–Lys225, Asp171–Lys225, Arg179–Asp212, Phe190–Arg301, Asp198–Lys236, Glu200–Lys309, Leu205–Ser260, Phe206–Arg248, and Gln209–Arg258. Helices α4–α5 and the bilobed core show greater structural correspondence among representative phenuivirus NPs (Figure 1F), whereas the distal N-terminal region is more variable. This distribution positions the core-associated helical module at the modeled oligomeric interface, whereas the divergent distal region lies largely outside the interface. These predicted interface assignments, derived entirely from the AF3 model, await direct experimental validation.

Progressive N-terminal truncations were designed from the AF3-derived domain boundaries (Figure S2B,C). Only NP_152–440_ yielded enough soluble protein for purification and further analysis. This construct lacked the lower-confidence distal N-terminal region and helices α1–α2 but retained helices α3–α5 and the complete bilobed core. Extending the deletion to residue 178 removed additional residues within the retained N-terminal helical region; NP_178–440_ did not produce sufficient soluble protein for downstream characterization under the tested conditions. Purified NP_152–440_ eluted as a predominant SEC peak and migrated at the expected molecular mass on SDS-PAGE (Figure 3E(a)). Absorbance at 260 nm remained higher than that at 280 nm, preserving the nucleic-acid-associated chromatographic signature of the full-length protein. Negative-stain electron microscopy identified ring-like NP_152–440_ particles (Figure 3E(b)). Deletion of residues 1–151 therefore did not abolish ring-like particle formation under the tested conditions. Within the retained region, the AF3 model predicted contacts between Trp165-Thr181, Ala166-Leu185, Gly162-Leu185, Leu163-Arg185, Phe159-Leu185, Phe159-Arg188, and Phe159-Ser189 (Figure 3F). NP_152–440_ yielded sufficient soluble protein for purification, whereas NP_178–440_ did not, precluding further characterization of the latter construct.

### 3.4. A Predicted Electropositive Groove Represents a Candidate Ligand-Interaction Surface in TcTV2 NP

Electrostatic surface analysis of representative phenuivirus NP–RNA complexes revealed a conserved electropositive groove between the two structural lobes of SFTSV, RVFV, and TOSV NPs, with RNA following a similar trajectory in each structure (Figure 4A). Superposition of the SFTSV NP–RNA complex onto the TcTV2 NP core showed that the SFTSV-associated RNA could be accommodated within the corresponding electropositive groove of TcTV2 NP without substantial steric overlap (Figure S3A). Representative NPs from *Nairoviridae*, *Arenaviridae*, *Hantaviridae*, and *Peribunyaviridae* exhibited distinct RNA-associated topologies and electrostatic surface distributions (Figure S3B). Structural divergence among bunyaviral NPs was uneven, whereas SFTSV, RVFV, and TOSV retained a similarly positioned electropositive RNA-binding groove. Suramin, a polyanionic compound previously reported to interact with the RNA-binding region of SFTSV NP, was used as a model ligand to probe the corresponding surface in TcTV2 NP [19]. Across three independent experiments, the apparent Tm was 46.97 ± 0.08 °C for TcTV2 NP alone and 39.25 ± 0.74 °C in the presence of suramin (mean ± SD), corresponding to a mean ΔTm of −7.72 ± 0.66 °C (Figure 4C), indicating altered protein thermal behavior rather than ligand-induced stabilization under the conditions tested. This negative thermal shift does not by itself establish direct or site-specific binding. Biolayer interferometry recorded concentration-dependent fitted responses across the 1.25–20 μM suramin series and yielded apparent *K_D_* values of 11.6 and 12.4 μM in two independent experiments (Figure 4D). These measurements support a possible association between suramin and recombinant TcTV2 NP.

The top-ranked docking pose (GlideScore = −11.056) was located near the predicted electropositive groove of the TcTV2 NP bilobed core (Figure 4E,F). Phe211, Asn251, Lys278, Ala280, Val281, and Arg290 surrounded the docked ligand and contributed predicted polar and hydrophobic contacts. During the single 100-ns molecular dynamics simulation, suramin remained associated with a region near the predicted electropositive groove (Figure 4G). After initial equilibration, protein and protein–ligand complex RMSDs remained predominantly within approximately 0.2–0.3 nm. Ligand RMSD increased early in the trajectory and subsequently fluctuated around 0.6 nm, consistent with conformational rearrangement of the flexible suramin molecule while maintaining association with this region. The representative post-simulation structure showed an extended network of polar, charged, and hydrophobic contacts along the groove (Figure 4H). Gln209 and Lys254 showed the highest calculated hydrogen-bond occupancies, with additional contributions from Asn251, Gln331, Lys388, Arg258, and Lys278 (Figure S3C). These occupancies describe contact persistence within a single modeled trajectory and do not identify experimentally validated binding determinants. The biophysical measurements support a possible association between suramin and recombinant TcTV2 NP under the conditions tested, whereas the docking and simulation analyses suggest a possible binding mode near the predicted electropositive groove. This groove represents a candidate ligand-interaction surface, although the present data do not establish the precise binding geometry or antiviral activity.

## 4. Discussion

TcTV2 is a divergent member of the genus *Uukuvirus*, yet its NP retains several features shared with better-characterized phenuivirus NPs. The predicted structure contains a bilobed core with an electropositive groove, recombinant TcTV2 NP associates with RNA during bacterial expression; and electron microscopy resolves several ring-like assembly classes. Deletion of residues 1–151 did not abolish ring-like particle formation under the conditions tested, while NP_152–440_ retained soluble expression and formed ring-like particles. Biophysical measurements support a possible association between suramin and recombinant TcTV2 NP under the conditions tested.

NPs within the order *Bunyavirales* share the task of viral RNA encapsidation but differ markedly in fold, RNA-binding topology, and oligomeric organization. Structures from the families *Peribunyaviridae*, *Nairoviridae*, *Hantaviridae*, *Arenaviridae*, and *Phenuiviridae* illustrate the range of NP architectures compatible with RNP formation [31,32,33,34,65,66,67]. A recent SAXS and AlphaFold 3 study of SGLV and BJNV NPs similarly found that a lineage-associated fold can persist despite substantial sequence divergence, whereas peripheral regions and RNP organization vary more extensively [68]. Within this structural spectrum, the predicted bilobed core of TcTV2 NP superimposes closely on the corresponding regions of SFTSV, RVFV, and TOSV NPs despite limited and uneven sequence conservation. Early structural work on RVFV NP identified the characteristic bilobed fold and a charged cleft implicated in RNA binding, and subsequent NP–RNA structures established that viral RNA occupies the groove between the two lobes [16,17,18]. TOSV and SFTSV NP structures exhibit comparable core organization and RNA accommodation [24,69]. The predicted TcTV2 NP model is consistent with this pattern, with structural similarity concentrated in the putative RNA-binding core and greater variation in the distal N-terminal and other peripheral regions. This organization agrees with comparative analyses showing that predicted NP structures can retain evolutionary relationships that are difficult to discern from primary sequences alone [36]. These structural interpretations remain model-derived. The low prediction confidence of the distal N-terminal region limits conclusions regarding its conformation, and the AF3-predicted pentameric arrangement and interprotomer contacts require experimental validation.

Electron microscopy revealed multiple ring-like particle classes formed by recombinant TcTV2 NP under the tested conditions. Among the 1990 classified particles, 1189 were pentamer-like, 492 were hexamer-like, 205 were heptamer-like, and 104 remained undefined. The predominance of pentamer-like particles does not obscure the coexistence of several discrete morphologies in the same recombinant preparation. Comparable variation in NP stoichiometry has been reported for other phenuivirus and bunyavirus proteins, both in structural studies and in analyses of coexisting RNA–protein complexes [18,25,70,71]. Such heterogeneity is compatible with the organization of bunyaviral RNPs, which must package RNA while remaining accessible to the viral polymerase during transcription and replication [8,10,72,73]. UUKV RNPs were previously observed as circular structures, whereas more recent studies have described circular, helical, and locally variable RNP arrangements in other bunyaviruses [72,74,75]. RNA accessibility may also differ along an encapsidated genome segment, as demonstrated for the RVFV S segment [76]. Because particle picking relied on low-pass-filtered RVFV NP references after the template-free approach failed to yield interpretable classes, enrichment of reference-like ring particles cannot be excluded. The TcTV2 particle classes therefore represent plausible in vitro manifestations of the structural variability available of NP–RNA assemblies, although their frequencies describe the analyzed particle dataset rather than an oligomeric equilibrium in solution.

The truncation experiments showed that NP152–440, which lacks residues 1–151 in the distal N-terminal region, remained soluble, retained the nucleic-acid-associated chromatographic profile, and formed ring-like particles. Thus, deletion of residues 1–151 did not abolish ring-like particle formation under the tested conditions. Because full-length NP and NP_152–440_ were not subjected to a matched quantitative comparison, these data do not establish whether deletion of residues 1–151 altered the distribution of particle morphologies. The NP178–440 construct, which removed an additional 26 residues, did not yield sufficient soluble protein for comparable analysis. The truncation data therefore show that NP_152–440_ retains the capacity to form soluble ring-like particles despite deletion of the distal N-terminal region.

RNA association provides an experimental observation that can be considered alongside the predicted RNA-binding core of TcTV2 NP. Full-length TcTV2 NP co-purified predominantly with RNA, and most recovered reads mapped to protein-coding regions of the *E. coli* genome. Removal of much of the associated nucleic acid reduced the A260/A280 ratios of both SEC species without substantially changing their relative chromatographic distribution. RNA therefore contributes to the absorbance profile but does not fully account for the two protein species. The association with bacterial transcripts demonstrates that recombinant TcTV2 NP can engage RNA in a heterologous cellular environment; it does not establish selective recognition of TcTV2 genomic RNA. When considered alongside the predicted electropositive groove and its alignment with phenuivirus NP-RNA structures, these biochemical observations are consistent with an RNA-association surface in TcTV2 NP.

The predicted electropositive groove also offers a candidate surface for examining ligand engagement. Suramin is particularly informative in this context because an SFTSV NP–suramin structure located part of the ligand within the putative RNA-binding cavity, and the same study reported effects on SFTSV NP–RNA binding and viral replication [19]. In the present study, across three independent experiments, the apparent Tm was 46.97 ± 0.08 °C for TcTV2 NP alone and 39.25 ± 0.74 °C in the presence of suramin (mean ± SD, n = 3), corresponding to a mean ΔTm of −7.72 ± 0.66 °C. This negative thermal shift reflects altered thermal behavior rather than ligand-induced stabilization and does not by itself establish direct or site-specific binding. Alternative explanations include protein destabilization or aggregation, dye-related effects, and changes in the NP–RNA complex. BLI produced concentration-dependent fitted responses over 1.25–20 μM, with apparent KD values of 11.6 and 12.4 μM in two independent experiments. Given the available controls, these measurements support a possible association between suramin and recombinant TcTV2 NP under the conditions tested but do not establish binding specificity or localize the interaction site. Docking and the single 100-ns MD trajectory provide a model-derived structural interpretation: suramin remains associated with a surface near the predicted electropositive groove and samples contacts involving several charged and polar residues, including Gln209 and Lys254. The computational model can now be tested by mutagenesis, RNA-competition assays, or structural analysis of an NP-ligand complex.

TcTV2 NP consequently combines a predicted phenuivirus-like RNA-binding core with experimentally observed RNA association and heterogeneous ring-like assembly. The ability of NP_152–440_ to form ring-like particles shows that deletion of residues 1–151 does not abolish ring formation under the tested conditions, although the effect of this deletion on particle distributions remains unresolved. Biophysical measurements support a possible association between suramin and recombinant TcTV2 NP under the conditions tested, whereas docking and a single 100-ns molecular dynamics simulation suggest the predicted electropositive groove as a candidate interaction surface.

## 5. Conclusions

In this study, we characterized the structural organization of TcTV2 NP, a divergent uukuvirus nucleoprotein within *Phenuiviridae*. The predicted TcTV2 NP model contains a phenuivirus-like bilobed architecture and an electropositive groove associated with phenuivirus RNA interaction. Recombinant TcTV2 NP forms heterogeneous ring-like assemblies, and deletion of residues 1–151 did not abolish ring-like particle formation under the conditions tested. Biophysical measurements support a possible association between suramin and recombinant TcTV2 NP, whereas docking and a single 100-ns molecular dynamics simulation suggest the predicted electropositive groove as a candidate interaction surface. These findings expand the structural understanding of divergent phenuivirus NPs and provide a framework for investigating the relationship between conserved RNA-associated features and heterogeneous assembly.

## Figures and Tables

**Figure 1:**
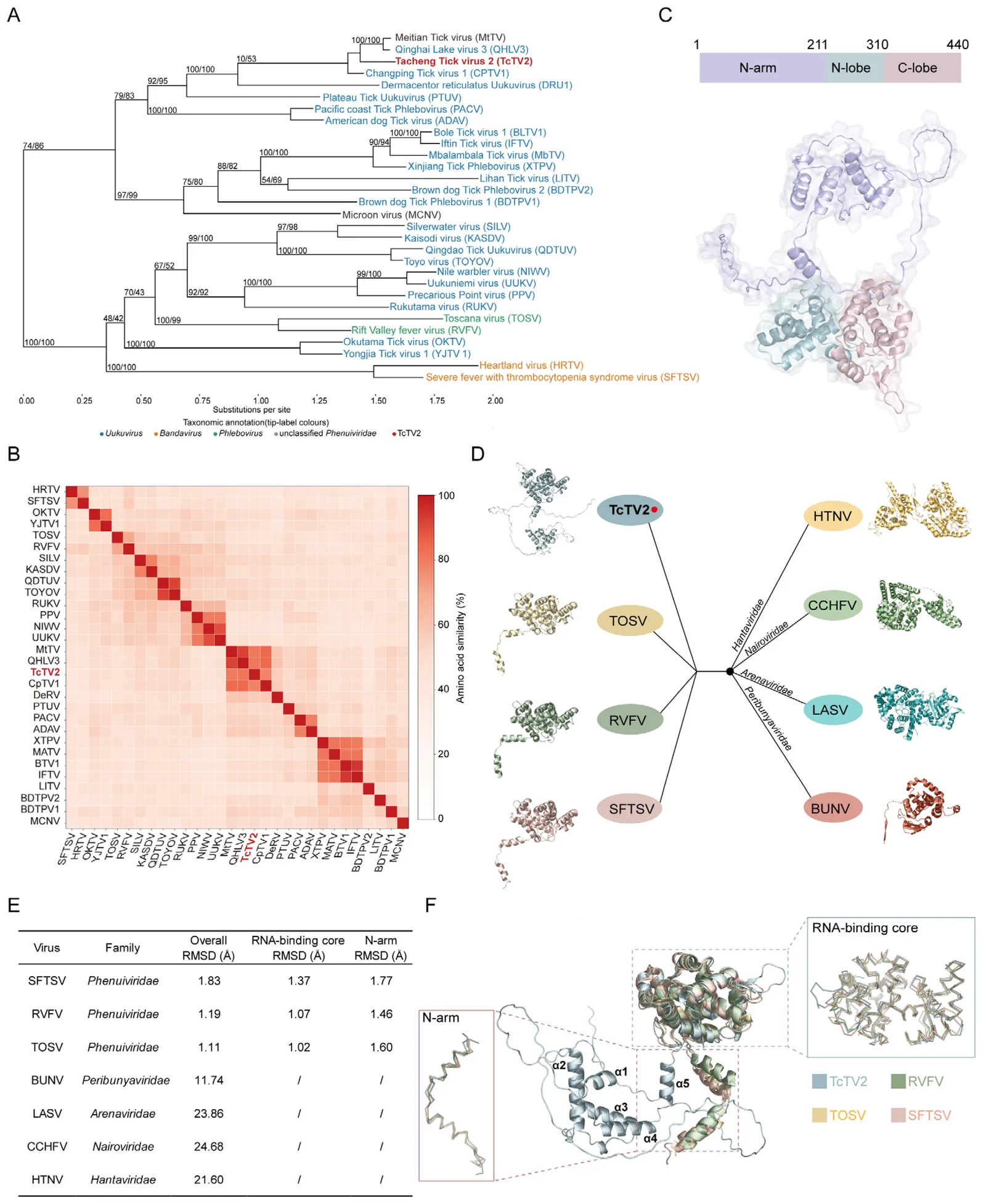
Sequence and predicted structural organization of TcTV2 NP. (**A**) Maximum-likelihood phylogeny inferred from 30 phenuivirus NP amino-acid sequences using 293 alignment positions retained after removal of columns with less than 50% non-gap occupancy. Node labels indicate SH-aLRT/ultrafast bootstrap support (%), each based on 1000 replicates. The tree was midpoint-rooted for graphical display, and branch lengths represent substitutions per site. TcTV2 is highlighted in red. (**B**) Pairwise NP amino acid similarity matrix calculated from the complete, untrimmed multiple-sequence alignment. Similarity was defined as the percentage of identical or BLOSUM62-positive residue pairs among positions containing residues in both sequences. TcTV2 is highlighted in red. (**C**) Domain organization and structural views of the full-length TcTV2 NP model predicted by the AlphaFold Protein Structure Database (AF-0000000365807691). (**D**) Representative NP structures from different viral families, including SFTSV (PDB: 9UMZ), RVFV (PDB: 4H5M), TOSV (PDB: 4CSG), BUNV (PDB: 3ZLA), LASV (PDB: 3MWP), CCHFV (PDB: 4AKL), and HTNV (PDB: 6I2N), together with the predicted TcTV2 NP model. (**E**) Cα RMSDs from overall and region-specific structural alignments. Detailed residue selections are provided in Table S2. (**F**) Superposition of TcTV2, SFTSV, RVFV, and TOSV NPs, showing greater structural similarity within the predicted RNA-binding core, including helices α4–α5, than in the peripheral N-terminal regions.

**Figure 2:**
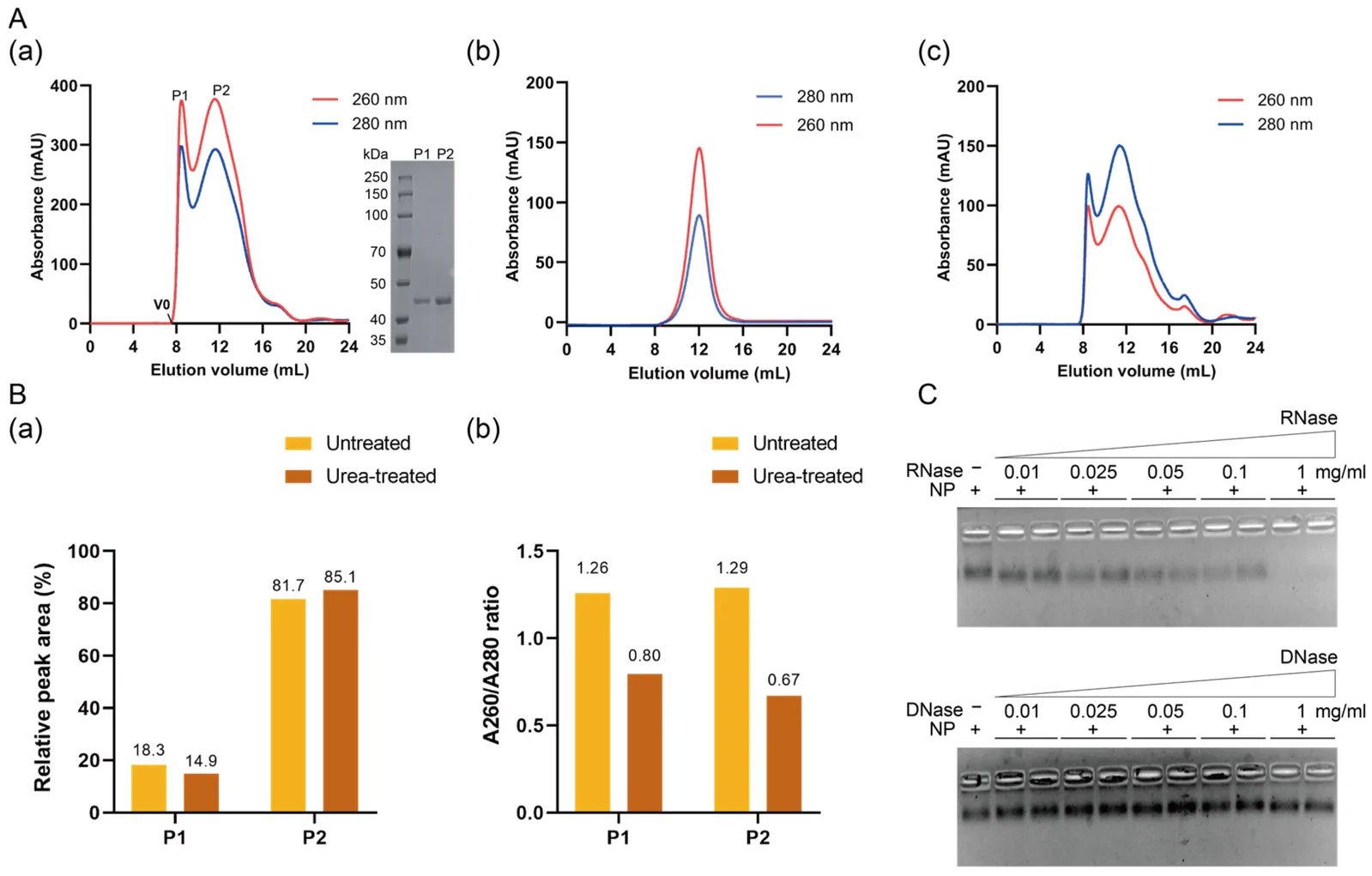
Recombinant TcTV2 NP co-purifies predominantly with RNA. (**A**) SEC profiles of full-length TcTV2 NP monitored at 260 and 280 nm. (**a**) Initial separation into partially resolved P1 and P2 peaks; inset, SDS-PAGE analysis of indicated fractions. (**b**) Rechromatography of the P2 fraction. (**c**) SEC profile after on-column urea denaturation and refolding. V0, void volume. (**B**) Comparison of untreated and urea-treated samples. (**a**) Relative peak areas of P1 and P2. (**b**) Peak-apex A260/A280 ratios. (**C**) Agarose gel analysis of NP-associated nucleic acids after treatment with RNase A or DNase I at the indicated concentrations. Untreated samples were used as controls.

**Figure 3:**
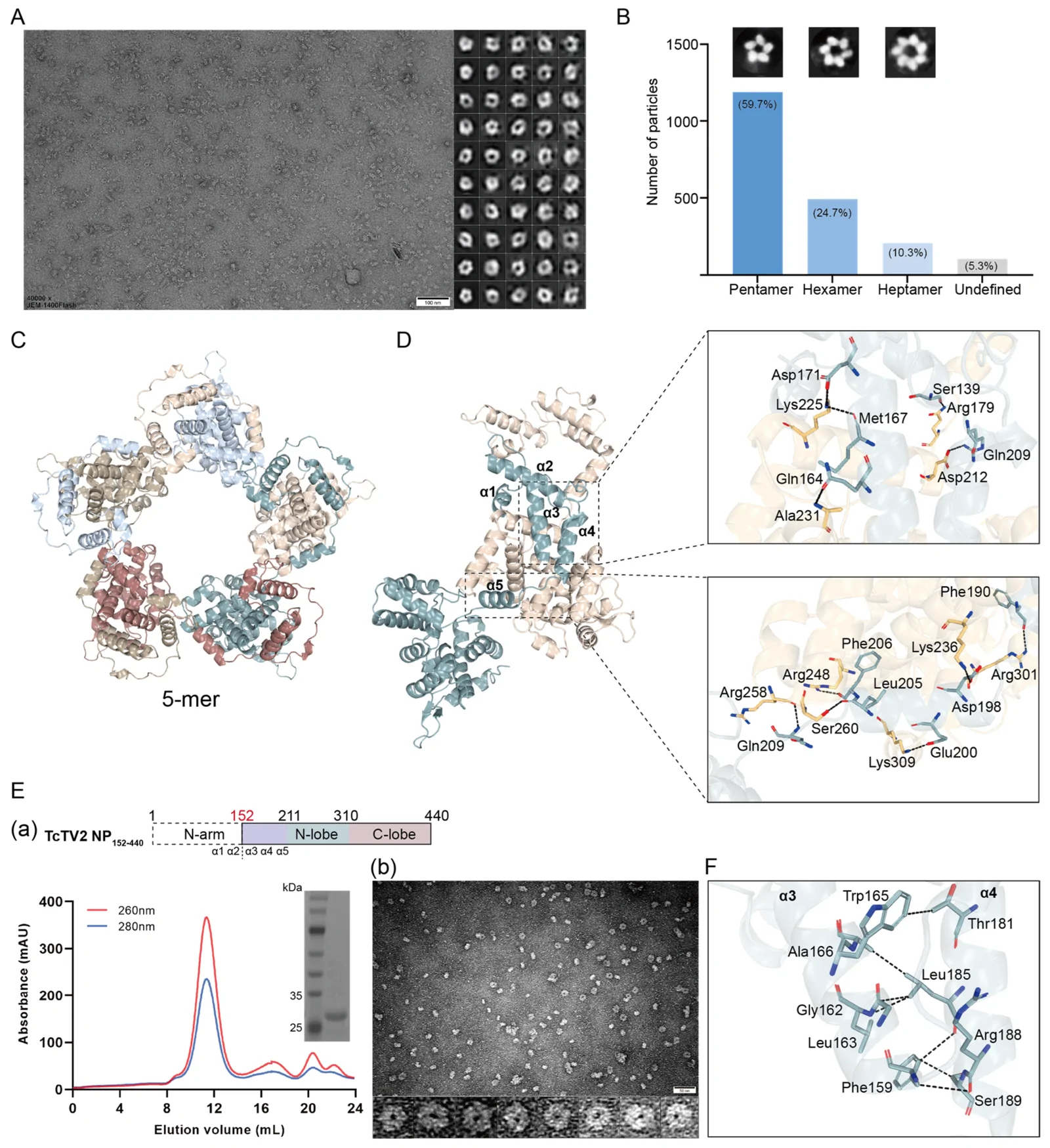
Heterogeneous ring-like particles formed by full-length and truncated TcTV2 NP. (**A**) Representative negative-stain electron micrograph of full-length TcTV2 NP and selected class averages. Scale bar, 100 nm. (**B**) Distribution of pentamer-like, hexamer-like, heptamer-like, and undefined particles from the cryo-EM dataset, with representative class averages shown above. (**C**) AlphaFold 3-predicted pentameric TcTV2 NP model. Residues 1–100 were omitted from visualization because of low prediction confidence. Individual NP protomers are shown in different colors for clarity. (**D**) Predicted interprotomer contacts between adjacent TcTV2 NP chains. The two interacting protomers are shown in teal and beige, respectively, with selected interacting residues shown as sticks. Oxygen and nitrogen atoms are shown in red and blue, respectively. (**E**) Characterization of NP_152–440_. (**a**) Construct design, SEC profile, and SDS-PAGE analysis. (**b**) Representative negative-stain electron micrograph and selected ring-like particles. Scale bar, 50 nm. (**F**) Predicted intramolecular contacts between helices α3 and α4 in NP_152–440_. he protein backbone is shown in light teal, and selected interacting residues are shown as sticks. Carbon, oxygen, and nitrogen atoms are shown in teal, red, and blue, respectively. Dashed lines indicate predicted intramolecular contacts.

**Figure 4:**
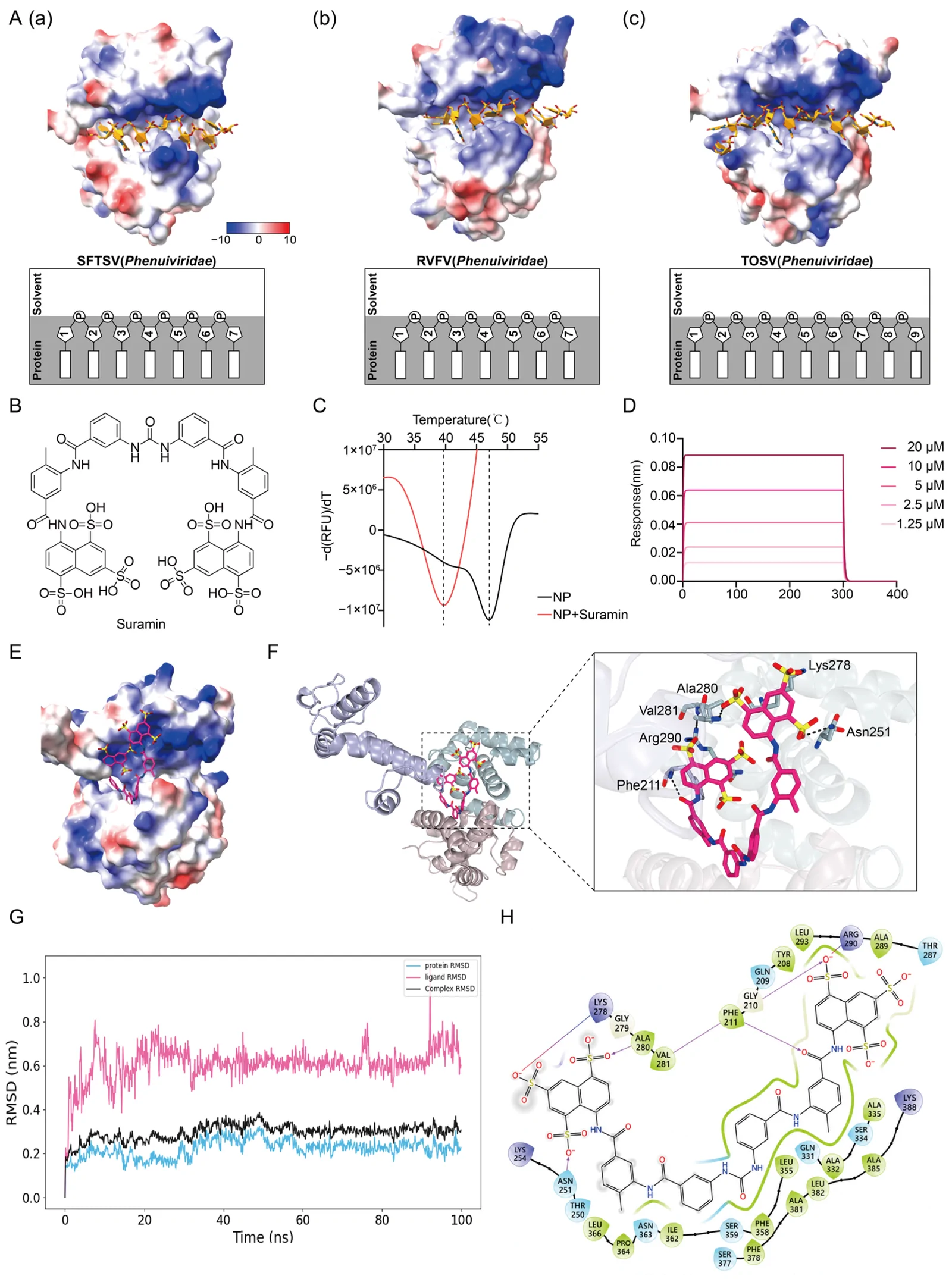
Conserved phenuivirus NP RNA-binding grooves and suramin association with TcTV2 NP. (**A**) Electrostatic surface views of RNA-bound NPs from (**a**) SFTSV (PDB: 9UMZ), (**b**) RVFV (PDB: 4H5P), and (**c**) TOSV (PDB: 4CSF). Surface electrostatic potential is colored from blue (positive) to red (negative), with white representing near-neutral regions. RNA is shown as orange sticks. The schematic diagrams below indicate the RNA path relative to the protein surface; numbers indicate the order of nucleotides in the bound RNA, and “P” denotes the phosphate group in the RNA backbone. (**B**) Chemical structure of suramin. (**C**) Thermal shift assay (TSA) curves of TcTV2 NP with or without suramin. Dashed lines indicate apparent melting temperatures. (**D**) Biolayer interferometry (BLI) responses of TcTV2 NP to 1.25–20 μM suramin after reference subtraction. (**E**,**F**) Electrostatic surface and cartoon views of the TcTV2 NP–suramin docking model. Labeled interactions are predicted contacts. (**G**) RMSDs of protein, ligand, and complex during the 100-ns molecular dynamics simulation. (**H**) Two-dimensional interaction map of a representative TcTV2 NP–suramin complex from the MD trajectory. Residues are colored by physicochemical property: positively charged, purple; polar, cyan; hydrophobic, green; and glycine, pale yellow. Magenta arrows indicate hydrogen bonds, red–blue lines indicate salt bridges, and gray halos indicate solvent exposure.

## Data Availability

Data are contained within the article and Supplementary Materials.

## Supplementary Materials

The following supporting information can be downloaded at https://www.mdpi.com/article/10.3390/life16101605/s1, Figure S1: Multiple-sequence alignment of representative phenuivirus nucleoproteins; Figure S2: Supporting cryo-EM, model-confidence, and truncation analyses of TcTV2 NP; Figure S3: Structural comparison of *phenuivirus* RNA-binding grooves and TcTV2 NP–suramin interactions. Table S1: Viral nucleoprotein sequences used for phylogenetic and pairwise amino-acid similarity analyses; Table S2: Structures and structural-alignment parameters used for comparison with the predicted TcTV2 nucleoprotein model; Table S3: Descriptive sequencing summary of RNA recovered with recombinant TcTV2 NP; Table S4: Confidence metrics for the AlphaFold-predicted monomeric and pentameric TcTV2 NP models; Table S5: Model-predicted intersubunit hydrogen bonds and salt bridges at the A–B interface of the TcTV2 NP pentamer; Table S6: Predicted TcTV2 NP residues interacting with suramin during molecular dynamics simulation.
