# Supplementary material for: Structural Conservation and Assembly Plasticity of a Divergent Phenuivirus Nucleoprotein
Source: Life (Basel). 2026 Sep 25;16(10):1605. doi: 10.3390/life16101605 (PMC13706394; doi:10.3390/life16101605)
Supplement: Supplementary file 1 [file life-16-01605-s001.zip › Supplementary_Figures_Revision.pdf]

Supplementary Figures

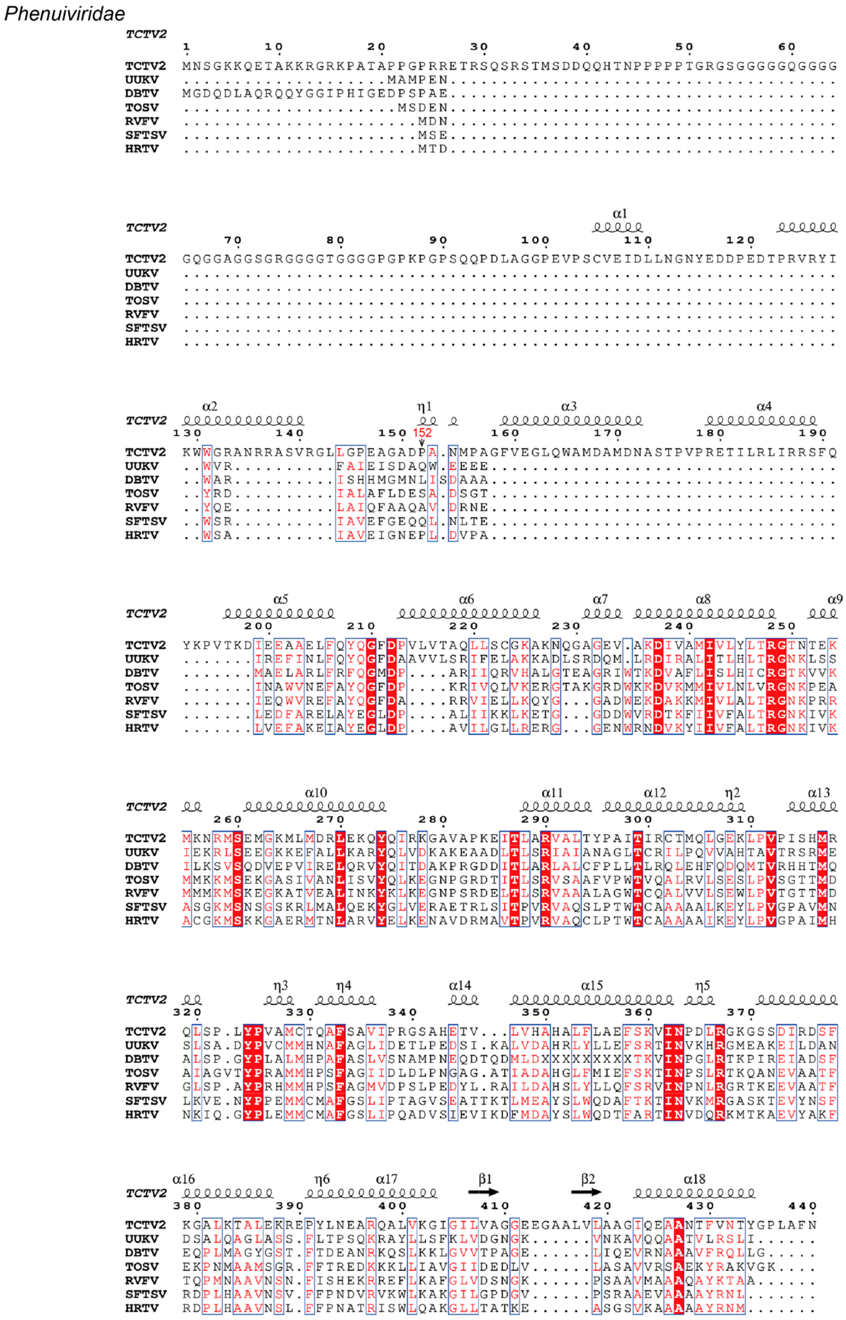

Figure S1. Multiple-sequence alignment of representative phenuivirus nucleoproteins. Multiple-sequence alignment of TcTV2, UUKV, DBTV, TOSV, RVFV, SFTSV, and HRTV NPs generated using MEGA v11 and rendered using ESPript 3. This representative subset was used for structural comparison and visualization and was not used for phylogenetic inference or calculation of the pairwise sequence-similarity values shown in Figure 1B. TcTV2 residue numbering and secondary-structure elements derived from the TcTV2 NP model are shown above the alignment. Identical and similar residues are indicated according to the ESPript 3 coloring scheme.

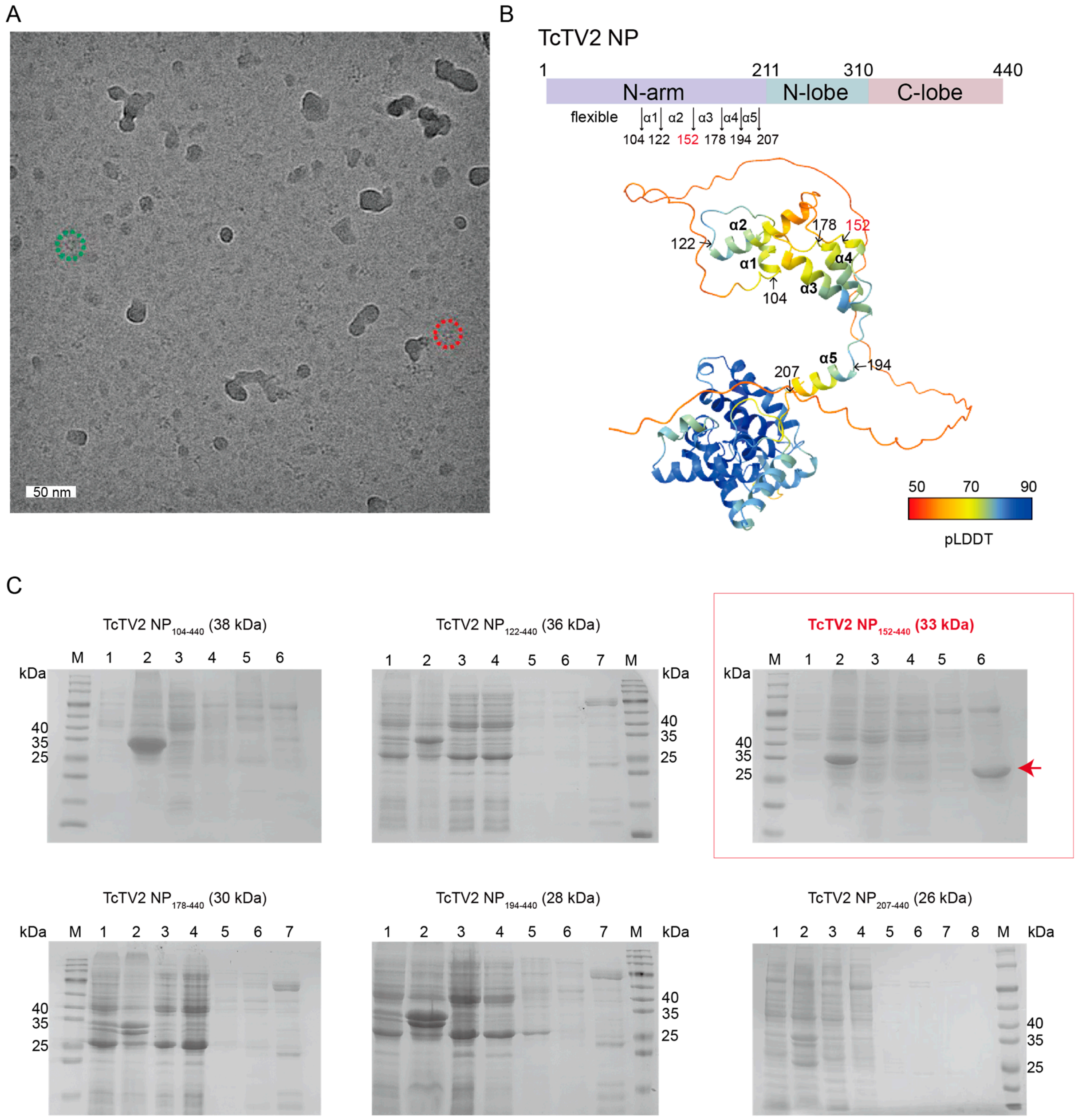

Figure S2. Supporting cryo-EM, model-confidence, and truncation analyses of TcTV2 NP. (A) Representative cryo-EM micrograph of full-length TcTV2 NP. Colored dashed circles indicate representative particle-like features. Scale bar, 50 nm. (B) Domain organization and AlphaFold Protein Structure Database monomeric model of TcTV2 NP colored according to pLDDT scores. The low-confidence region and helices α1–α5 are indicated, and residue 152 is highlighted in red.(C) SDS-PAGE analysis of expression, solubility, and Ni-NTA purification of TcTV2 NP truncation constructs ($NP_{104-440}$, $NP_{122-440}$, $NP_{152-440}$, $NP_{178-440}$, $NP_{194-440}$, and $NP_{207-440}$). Lanes 1-7 represent total lysate, insoluble fraction, flow-through, wash fractions (20-100 mM imidazole), and elution fractions (500 mM imidazole), respectively. $NP_{152-440}$, selected for further purification and characterization, is highlighted.

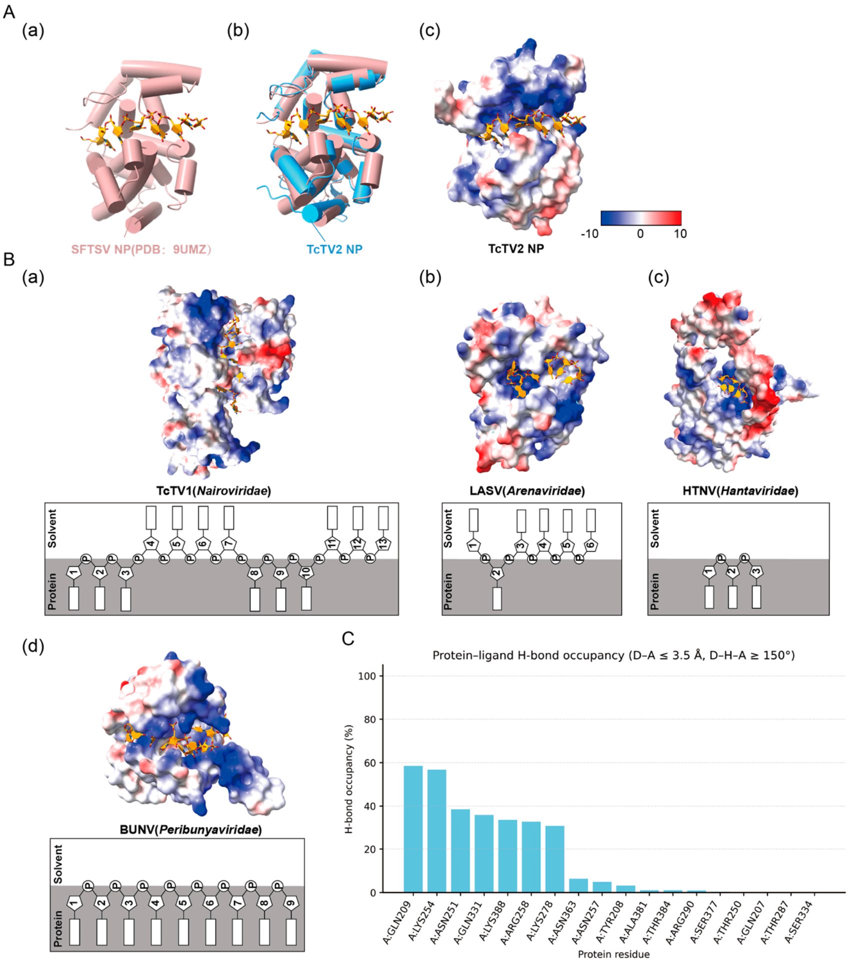

Figure S3. Structural comparison of *phenuivirus* RNA-binding grooves and TcTV2 NP–suramin interactions. (**A**) (a) SFTSV NP–RNA structure (PDB: 9UMZ, chain C), (b) superposition of the TcTV2 NP model with SFTSV NP, and (c) electrostatic surface of TcTV2 NP with the SFTSV-derived RNA coordinates superimposed for visualization. (**B**) Electrostatic surface representations and schematic RNA-binding topologies of representative (a) CCHFV (*Nairoviridae*; PDB: 4AKL), (b) LASV (*Arenaviridae*; PDB: 3T5N), (c) HTNV (*Hantaviridae*; PDB: 6I2N), and (d) BUNV (*Peribunyaviridae*; PDB: 3ZLA) NPs. Surfaces are displayed using a common electrostatic-potential scale, with negatively and positively charged regions shown in red and blue, respectively. (**C**) Protein-suramin hydrogen-bond occupancies calculated from the 100-ns molecular dynamics trajectory. Hydrogen bonds were defined using a donor–acceptor distance ≤3.5 Å and a D–H···A angle ≥150°. Residue labels include the protein chain identifier A.
